# Jubilee of the Medical Department, University of Buffalo

**Published:** 1896-04

**Authors:** 


A Monthly Review of Medicine and Surgery.

*EDITORS:*

THOMAS LOTHROP, M. D. - - WM. WARREN POTTER, M. D.

All communications, whether of a literary or business nature, should be addressed to the managing editor: 284 FRANKLIN STREET, BUFFALO, N. Y.

VOL. XXXV. APRIL, 1896. No. 9.

### JUBILEE OF THE MEDICAL DEPARTMENT, UNIVERSITY OF BUFFALO.

THE medical profession of Buffalo is just now passing through a series of anniversaries. Last August the JOURNAL celebrated its semi-centennial by issuing a jubilee number that has received everywhere the commendation of our contemporaries. In January of this year the Medical Society of the County of Erie reached the seventy-fifth year-post of its life, which was appropriately accentuated by anniversary proceedings that already have been recorded in these pages. Now comes the Medical Department of the University of Buffalo reminding us that it has reached the half-century mark and that it proposes to commemorate the event by more than the ordinary ceremonies at its forthcoming annual commencement, that is fixed for May 5, 1896.

We are not informed as to the precise scope of these special observances, but believe that, among other things, an appeal is making to the alumni to come and attend the feasts, both intellectual and material, that their alma mater is preparing. It would seem appropriate that her children should gather and make merry on this extraordinary occasion, and we presume they will do so in large numbers.

In our jubilee number (August, 1895,) we gave a historical sketch of this institution, but in view of the event above alluded to it seems appropriate that we should refer again to some of the principal data connected with the history of the college.

The changes which have taken place since 1846 are many and noteworthy, but concern the medical department alone, inasmuch as until 1884 this was the only department in existence. Its first home was in a church building upon the site of the present post-office and afterward in the old stone edifice on the corner of Virginia and Main streets, where it remained until 1893. It is within the memory of all how inadequate and imperfect in many ways was this structure for the purposes of modern medical teaching. There were but few teachers, a meager and ridiculously abbreviated course, no laboratories and poor equipment for very many years.

And yet the teachers, who were, so to speak, professional giants, did wonderfully good work in the light of those days. They were, indeed, in many respects in advance of their own period; or, at all events, were fully abreast of it in every way. Little by little improvement was made, until today we find the department occupying a well-appointed building, as handsome and as well designed for its purpose as any in the land. Its corps of instructors has increased from seven to fifty-seven, its course embracing every branch recognised as essential for a complete medical education, including laboratory instruction in biology, histology, pharmacy, chemistry and pathology, all of which are obligatory and thoroughly taught in spacious well-equipped laboratories. Clinics and ward classes are held daily in the practical branches, the students being thus brought into immediate contact with disease in its many forms.

One of the last acts of the medical faculty was to make a four years' course obligatory upon all future students. This will apply to all students entering in September, 1896. In every way the department has tried to aid the cause of higher medical education and today in this respect it stands at the fore with the leading medical colleges in the country.

Since 1884 a department of pharmacy, of law, of dentistry and of pedagogy have been added, all of which have successfully striven to uphold the reputation and worth of the university. There are now over seven hundred students in the several departments who have heartily coöperated with the officers and teachers to give name and fame to the institution. It will doubtless be the policy of the council of the university to create from time to time such additional departments as may be needed, and it is not too much to predict that the future will see in Buffalo a university that will rank with those now existing in other parts of the land.

The approaching commencement will be notable for its historic interest, as well as for its many interesting features. It should command a full attendance of the alumni.

